# Effectiveness of Evidence-Based Telecoaching Delivered Through an Employer-Sponsored Mental Health Benefits Platform

**DOI:** 10.1089/tmj.2020.0555

**Published:** 2022-04-19

**Authors:** Sara J. Sagui-Henson, Maximo R. Prescott, Julia B. Corcoran, Sanil Pillai, Lindsey Mattila, Somya Mathur, Terry Adkins, Myra Altman

**Affiliations:** ^1^ Modern Health, San Francisco, California, USA.; ^2^ Clinical Excellence Research Center, Stanford School of Medicine, Palo Alto, California, USA.

**Keywords:** telecoaching, mental health, well-being, burnout, presenteeism, workplace

## Abstract

**Introduction:**

Coaches delivering telemental health services as part of an employer-sponsored benefit may increase access to affordable and effective care. We examined the effectiveness of evidence-based telecoaching delivered via videoconferencing to people requesting mental health services during the coronavirus disease 2019 (COVID-19) pandemic.

**Materials and Methods:**

We analyzed data from 1,228 employees (mean age = 35 ± 8 years; 67.2% female) who utilized telecoaching through the Modern Health benefits platform between March 11, 2020 and March 11, 2021. We used paired samples t tests to examine changes in well-being, burnout, absenteeism, and presenteeism before and after telecoaching and moderated regressions to test whether these changes depended on visit utilization. We analyzed rates of clinical improvement for well-being and reduction from entry in symptoms for burnout. We conducted analyses in the full sample and participants presenting with elevated symptoms at baseline.

**Results:**

Participants utilized an average of 2.6 visits. Well-being (p = 0.02) significantly increased, while both presenteeism (p < 0.001) and absenteeism (p < 0.001) significantly decreased at follow-up in our full sample, but represented negligible effect sizes. Burnout was not found to have significantly changed at follow-up in our full sample (p = 0.69). In participants beginning care with elevated depressive-related symptoms, well-being significantly increased (p < 0.001) and 46.3% experienced a clinically relevant improvement. In participants beginning care with elevated levels of burnout, burnout significantly decreased (p < 0.001) and 20.9% experienced a reduction in symptoms from entry.

**Conclusions:**

Leveraging videoconferencing, telecoaching had positive effects on mental health and workplace outcomes, even during the COVID-19 pandemic. Evidence-based telecoaching represents a promising option for achieving optimal outcomes in people who need mental health services.

## Introduction

In 2016, mental illnesses affected nearly 1 billion people globally and accounted for 7% of disability-adjusted-life-years.^1^ Depression is a leading cause of disability,^2^ with nearly 1 in 5 U.S. adults experiencing a mental illness.^3^ The need for mental health services is pressing as the psychosocial consequences of the coronavirus disease 2019 (COVID-19) pandemic continue to manifest across the population. Nationally representative longitudinal studies in the United States have found alarming impacts on mental health,^4^ including a three-fold increase in the prevalence of depressive symptoms and 40% of adults reporting adverse behavioral health symptoms during the pandemic.^5,6^

Although rates of mental distress are higher in unemployed individuals, the increase in distress relative to previous trends has been greatest among individuals employed before the pandemic.^7^ Mental distress impacts the mental health of employees and results in lost productivity and subsequent economic costs for employees, employers, and society. Absenteeism (a pattern of being absent from work due to illness) and presenteeism (a pattern of working despite illness) are both negative indicators of productivity affected by a worker's mental health.^8,9^ Employers are increasingly interested in improving the mental health of their workforce to subsequently reduce absenteeism and presenteeism behavior. The World Health Organization (WHO) estimates that anxiety and depression cost the global economy $1 trillion each year in lost productivity, and that for every $1 spent on scaling mental health treatment, there is a $4 return in improved health and productivity.^10,11^

Although existing treatments such as cognitive behavioral therapy (CBT) are effective at addressing and improving mental well-being, more than 70% of persons who need mental health services lack access to care.^12^ The pandemic likely compounds this mental health treatment gap as both the need for mental health services has dramatically increased and it has disrupted opportunities to deliver care with direct patient encounters in clinical practice.^13^ One of the significant barriers to accessing mental health services is a shortage of highly trained mental health professionals able to provide treatment.^14^

A promising alternative is the activation of lower-intensity care delivered by bachelor's level trained providers, which may be effective in treating mental health challenges.^15,16^ Paraprofessionals are effective in delivering CBT to treat anxiety and depression, with comparable outcomes to those professionally trained with graduate degrees and more experience.^17^ Paraprofessionals practicing coaching may help individuals understand and work toward their mental health goals by improving self-efficacy and self-confidence,^18^ goal attainment, and metacognition.^19^ A meta-analysis of coaching found that receiving care from a coach enhanced subjective well-being, coping, and work attitudes.^20^ In the workplace, when managers engage in coaching, team members experience improved job satisfaction and work engagement.^21^

Furthermore, coaching interventions can positively impact distress, burnout, and life satisfaction.^22^ While evidence-based coaching has emerged as a promising intervention for improving well-being and work performance,^20^ the evidence on its effectiveness when delivered virtually in real-world workplaces is limited,^23^ and its effect on productivity outcomes is mixed.^22^ Real-world studies evaluating the impact of evidence-based telecoaching—virtually delivered coaching services—on mental health and workplace outcomes are urgently needed to demonstrate whether coaches can provide a scalable solution to help meet the unprecedented need for mental health services exacerbated by the COVID-19 pandemic.

### Study Overview

Using a retrospective cohort design, we analyzed pre-existing data collected from employees during routine care (i.e., chart review) who had initiated telecoaching through an employer-sponsored mental health benefit, Modern Health, during the COVID-19 pandemic. The primary aim of this analysis was to evaluate self-reported well-being, burnout, absenteeism, and presenteeism before and after telecoaching. We hypothesized that participants would report improvements in subjective well-being and reductions in burnout, absenteeism, and presenteeism. The secondary exploratory aims of this analysis were to evaluate changes in subjective well-being and burnout among participants with elevated baseline symptoms (depressive-related and burnout) and whether utilization moderated associated changes in outcomes. We hypothesized that telecoaching would be more effective with greater visit utilization and in those with elevated baseline depressive-related and burnout symptoms.

## Materials and Methods

### Participants

Participants were employees who completed telecoaching through Modern Health between March 11, 2020 and March 11, 2021 during the COVID-19 pandemic. Eligible participants were 18 years or older, had access to a smartphone, tablet, or computer, completed at least one assessment before telecoaching and one assessment after (see Measures section), and did not receive therapy through Modern Health during the study period. This study was reviewed by Aspire IRB and determined to be exempt from IRB oversight.

### Procedures

**Fig. 1.**
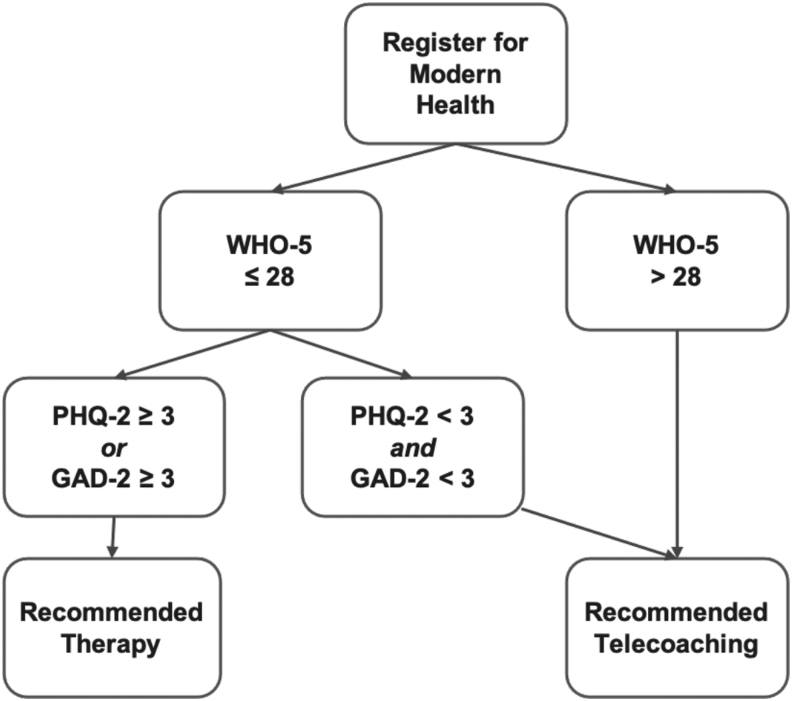
Care recommendation flow.

Employees registered for Modern Health on the web or mobile application using a device (smartphone, tablet, or computer) they had personal access to. At baseline, participants completed the WHO-5 well-being questionnaire; if they scored below the clinical cutoff for depressive symptoms (≤28), we administered the Patient Health Questionnaire-2^24^ and the Generalized Anxiety Disorder-2.^25^ See *Figure 1* for the recommended care flow. Participants who were recommended to therapy were still able to access telecoaching services if requested and coaches were trained to refer participants to therapy when needed. Participants also completed measures of burnout, absenteeism, and presenteeism. Next, participants selected a coach from a list provided by a matching algorithm and scheduled a visit. Telecoaching through Modern Health was an employee benefit provided by employers. There were no prescribed number of telecoaching visits to complete and participants could utilize telecoaching and complete assessments at their discretion. Participants could message with their coach between sessions and could rate their satisfaction after each visit. Follow-up assessments included the WHO-5, burnout, absenteeism, and presenteeism and were voluntarily completed any time through Modern Health's secure platform.

### Evidence-Based Telecoaching

All coaches were certified by an International Coaching Federation accredited program and screened by Modern Health to ensure that they were trained in and offered evidence-based approaches (techniques that draw from CBT, Acceptance and Commitment Therapy, Motivational Interviewing). Screening included reviewing responses to sample case studies, questions about coaches' theoretical framework, and a sample visit evaluation. Coaches received additional training in evidence-based approaches, including didactics and case consultation with a licensed therapist and certified coach. To ensure ongoing quality, Modern Health reviewed aggregate, anonymized employee feedback, which was provided after each visit. Coaching visits were 30-min long and provided via videoconferencing through Modern Health's platform. Coaches were not required to follow any single protocol during a telecoaching visit and were instead encouraged to leverage evidence-based techniques as they deemed appropriate to meet the unique needs of each individual.

### Measures

#### Well-being

We used the WHO-5^26^ to assess well-being within the past two weeks on a six-point scale (0 = at no time, 5 = all of the time), with higher summed scores indicating greater well-being. This is a unidimensional assessment of well-being with high clinimetric validity as a screening tool for depression.

#### Burnout

We used a validated single-item measure to assess the frequency of burnout.^27^ Participants responded to the item, “I feel burned out from my work,” on a seven-point scale (0 = never, 6 = every day). This item is used as a brief assessment that predicts outcomes with similar consistency as the full Maslach Burnout Inventory.^27^ High levels of burnout are defined as a frequency of “once a week” or more (≥4).

#### Absenteeism and presenteeism

We used validated single-item measures^28^ adapted from the Work Ability Index.^29^ Participants responded to the items, “In the past year, I missed X days because I was sick,” and “In the past year, I came to work X days even though I was sick,” on a seven-point scale (0 = none, 6 = more than 30 days) to measure absenteeism and presenteeism, respectively. Lower scores on both measures indicate greater productivity. Self-reported absenteeism measures have good psychometric properties, including adequate test-retest reliability and convergence with organizational records, and are used in large-scale epidemiological research and public surveys.^30–32^

#### Visit utilization

We assessed the number of visits each participant completed with a coach and used this as a categorical moderator variable (coded as 1 = 1 visit, 2 = 2–3 visits, and 3 = 4+ visits).

#### Satisfaction with care

Satisfaction ratings can serve as a proxy for treatment acceptability and therapeutic alliance. We assessed satisfaction with a 5-star rating. Modern Health prompts employees to rate their satisfaction after every coaching visit.

### Data Analysis

#### Data selection

We excluded participants with invalid data, defined as either having baseline and follow-up assessments collected more than 2 weeks before the first coaching visit or after the final coaching visit.

#### Data preparation

We performed analyses with R (Version 4.0.3) and SPSS (Version 25.0).^33^ We screened data for outliers, skewness, and kurtosis.^34^ Absenteeism and presenteeism scores were positively skewed. We square root transformed them which normalized the distributions for presenteeism (baseline: skewness = 0.22, kurtosis = 1.97; follow-up: skewness = 0.26, kurtosis = 1.84) and absenteeism (baseline: skewness = 0.08, kurtosis = 1.79; follow-up: skewness = 0.26, kurtosis = 1.85). Approximately 19.2% of cases were missing data and a Hawkins Test^35^ suggested that data were missing completely at random *p* = 0.09.

#### Pre-post changes in outcomes

We used two-tailed paired samples *t* tests comparing baseline and follow-up well-being, burnout, absenteeism, and presenteeism. To assess the impact of coaching on employees presenting with elevated levels of depressive-related symptoms, we performed *t* tests for individuals with baseline WHO-5 scores ≤28. We also performed *t* tests for individuals with elevated baseline burnout (≥4).

#### Visit utilization moderating changes in outcomes

We used moderated regression with the Mediation and Moderation for Repeated Measures^36^ macro for SPSS to test whether visit utilization moderated changes in each outcome. This analysis allows for the estimation of an interaction between a two-instance repeated-measures factor (pre- and post-well-being) and a single between-participant moderator (visit utilization).

#### Rates of clinically relevant change, reduction from entry, and treatment response

Clinically relevant change in well-being is defined as an increase of at least 10 points on the WHO-5.^26,37^ Treatment response for well-being is defined as a 50% increase in the WHO-5 score.^38^ Reduction from entry in burnout was defined as an individual's score changing from above (≥4) to below (<4) the cutoff for high burnout symptoms.^39^ We analyzed rates in the full sample and in those with elevated baseline depressive-related or burnout symptoms.

#### Satisfaction with care

We used descriptive statistics to analyze satisfaction ratings in the full sample and in participants with elevated baseline depressive or burnout symptoms.

## Results

### Participant Characteristics and Descriptive Statistics

**Fig. 2.**
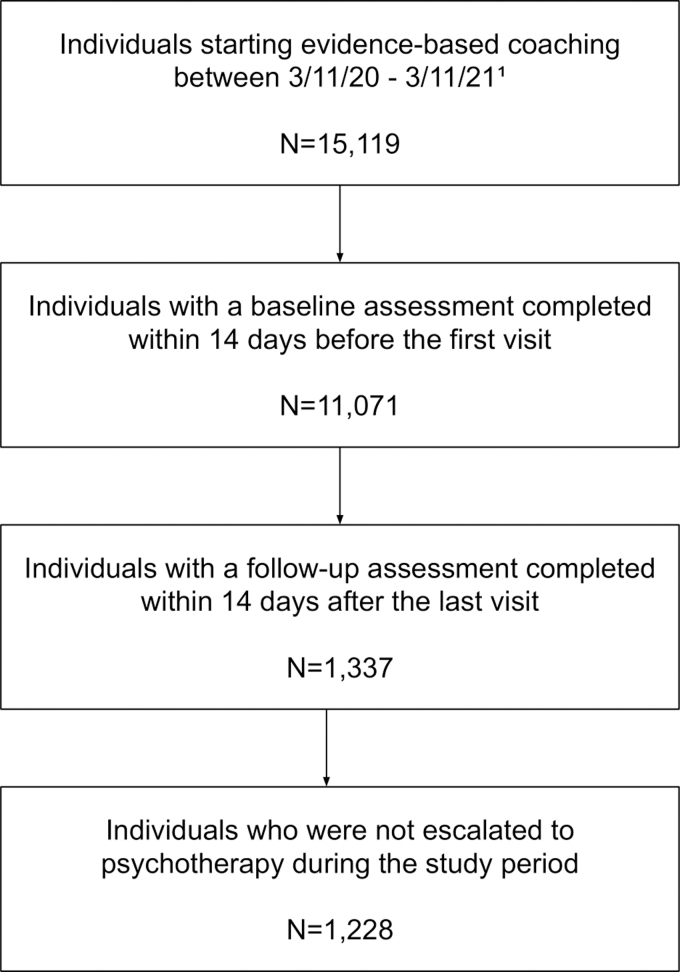
Participant flow. ^1^The 13,891 participants who did not provide valid assessments utilized an average of 2.7 (SD = 2.9) telecoaching visits during the study period. SD, standard deviation.

Of the 15,119 participants who had initiated telecoaching, 1,228 met inclusion criteria (*Fig. 2*). Participants were employed across 174 companies. Telecoaching was delivered by 155 coaches, with each coach providing care to an average of 8 participants (standard deviation [SD] = 10.24). Participants utilized an average of 2.4 coaching visits (SD = 2.6) across an average of 28.1 days in care (SD = 54.4). Average time between baseline and follow-up assessments was 37.25 days (SD = 54.79).

Age and sex data were missing for a portion of the sample as this information has been optional for employers to include in eligibility files. Participants with age data (*n* = 961) were, on average, 34.83years old (SD = 8.45, range: 20–66). Of participants with sex data (*n* = 777), 67.2% (*n* = 522) identified as female and 32.8% (*n* = 225) identified as male. See *Table 1* for descriptive statistics.

**Table 1.** Descriptive Statistics for Focal Variables

VARIABLE	*N*	M (SD)	RANGE	SKEWNESS	KURTOSIS
Well-being					
Baseline	1,228	45.18 (16.77)	0–100	0.04	2.61
Follow-up	1,228	46.27 (18.84)	0–100	−0.002	2.46
Burnout					
Baseline	1,181	3.43 (1.67)	0–6	−0.14	2.09
Follow-up	1,185	3.42 (1.68)	0–6	−0.27	2.17
Absenteeism					
Baseline	1,169	1.04 (1.13)	0–6	1.37	5.32
Follow-up	1,124	0.94 (1.12)	0–6	1.57	5.96
Presenteeism					
Baseline	1,128	1.20 (1.40)	0–6	1.57	5.53
Follow-up	1,095	0.98 (1.25)	0–6	1.68	6.13

Descriptive statistics are presented for all raw variables (i.e., before transformation).

SD, standard deviation.

### Pre-Post Changes in Outcomes

See *Table 2* for *t* test results. In our full sample, there was a statistically significant increase of an average of 1.09 points in well-being between baseline and follow-up (*p* = 0.02), but a negligible effect size (*d* = 0.06). Among participants with elevated baseline depressive-related symptoms, well-being significantly increased an average of 11.2 points on the WHO-5 (*p* < 0.001), representing a medium effect size (*d* = 0.81). There was no difference in burnout between baseline and follow-up (*p* = 0.69). Among those with elevated baseline burnout, burnout significantly decreased an average of 0.49 points (*p* < 0.000), with a medium effect size (*d* = 0.39). There was a statistically significant decrease of an average of -0.05 points in absenteeism between baseline and follow-up (*p* = < 0.001), but a negligible effect size (*d* = -0.08). Presenteeism significantly decreased an average of 0.09 points (*p* = 0.047), representing a negligible effect size (*d* = -0.12).

**Table 2.** Two-Tailed Paired *t* Test Results for Baseline and Follow-up Telecoaching Outcomes

OUTCOME & SAMPLE	*N*	BASELINE	FOLLOW-UP	PAIRED PRE-POST-DIFFERENCE	CI OF PRE-POST-DIFFERENCE	*t* (*df*)	*p*
		M (SD)	M (SD)	M (SD)	CI		
Well-being							
Full	1228	45.18 (16.77)	46.27 (18.84)	1.09 (16.23)	0.19 to 2.01	2.37 (1227)	0.02
Elevated depressive symptoms at BL (WHO-5 ≤ 28)	227	21.32 (6.87)	32.53 (16.62)	11.21 (15.45)	9.19 to 13.23	10.93 (226)	<0.001
Burnout							
Full	1151	3.43 (1.67)	3.42 (1.68)	0.016 (1.34)	−0.06 to 0.09	0.39 (1150)	0.69
Elevated burnout at BL (≥4)	536	4.95 (0.76)	4.46 (1.23)	−0.49 (1.15)	−0.59 to −0.39	−9.81 (535)	<0.001
Absenteeism							
Full	1089	0.77 (0.67)	0.70 (0.67)	−0.06 (0.52)	−0.09 to −0.02	−3.51 (1088)	<0.001
Presenteeism							
Full	1042	0.82 (0.73)	0.70 (0.70)	−0.09 (0.59)	−0.13 to −0.06	−5.00 (1041)	<0.001

Values for absenteeism and presenteeism represent the square root of original responses.

BL, baseline; CI, 95% confidence intervals; M, mean; WHO, World Health Organization.

### Visit Utilization Moderating Changes in Outcomes

See *Table 3* for moderated regression results. Visit utilization significantly moderated the difference between baseline and follow-up well-being. For every unit increase in the moderator, there was a 3.14 point increase in well-being. When participants completed 1 visit, their well-being did not change; when participants completed 2–3 visits, their well-being significantly increased by an average of 2.12 points; and when participants completed 4+ visits, their well-being significantly increased by an average of 5.27 points. Visit utilization did not moderate the difference between baseline and follow-up burnout (*b* = −0.006, standard error [SE] = 0.05, 95% confidence interval [−0.1 to 0.09], *R*^2^ = 0.003, *p* = 0.9). Visit utilization moderated the difference between baseline and follow-up presenteeism. For every 1 unit increase in the moderator, there was a 0.08 point decrease in presenteeism. When participants completed 1 visit, their presenteeism did not change; when participants completed 2–3 visits, their presenteeism significantly decreased by 0.11 points; and when participants completed 4+ visits, their presenteeism significantly decreased by 0.20 points.

**Table 3.** Moderated Regression Results for Visit Utilization Moderating the Difference in Outcomes Before and After Telecoaching

	WELL-BEING				PRESENTEEISM				ABSENTEEISM			
	*b*	SE	CI	*R* ^2^	*b*	SE	CI	*R* ^2^	*b*	SE	CI	*R* ^2^
Intercept	−4.16^^*^^	1.11	−6.33 to −1.99	0.02^^*^^	0.05	0.46	−0.04 to 0.14	0.01^^*^^	0.09^^*^^	0.04	0.01 to 0.16	0.01^^*^^
Visit utilization	3.14^^*^^	0.60	1.96 to 4.32		−0.08^^*^^	0.02	−0.13 to −0.04		−0.08^^*^^	0.02	−0.12 to −0.04	
Conditional effects												
1 Visit	−1.02	0.61	−2.22 to 0.18		−0.03	0.03	−0.08 to 0.02		−0.002	0.02	−0.04 to 0.04	
2–3 Visits	2.12^^*^^	0.50	1.15 to 3.10		−0.11^^*^^	0.02	−0.15 to −0.08		−0.08^^*^^	0.02	−0.11 to −0.05	
4+ Visits	5.27^^*^^	0.92	3.46 to 7.07		−0.20^^*^^	0.04	−0.27 to −0.13		−0.16^^*^^	0.03	−0.23 to −0.10	

Well-being *n* = 1,228. Presenteeism *n* = 1,042. Absenteeism = 1,089. *b* = unstandardized regression coefficient. The difference in each outcome was constructed by subtracting pre-telecoaching scores from post-telecoaching scores, with higher scores reflecting greater post-telecoaching well-being or presenteeism.

^^*^^p < 0.001.

SE, standard error.

Visit utilization moderated the difference between baseline and follow-up absenteeism. For every 1 unit increase in the moderator, there was a 0.08 point decrease in absenteeism. When participants completed 1 visit, their absenteeism did not change; when participants completed 2–3 visits, their absenteeism significantly decreased by 0.08 points; and when participants completed 4+ visits, their absenteeism significantly decreased by 0.16 points.

### Rates of Clinically Relevant Change, Reduction from Entry, and Treatment Response

Regarding rates of change, 25.6% of all participants and 46.3% of those with elevated baseline depressive-related symptoms experienced a clinically relevant increase in well-being. The treatment response rate for well-being was 14.3% in the full sample and 47.6% in those with elevated baseline depressive-related symptoms. Regarding burnout, 9.4% of all participants and 20.9% of those with elevated baseline burnout symptoms experienced a reduction from entry.

### Satisfaction with Care

Sixty-four percent (*n* = 785) of participants submitted at least one post-visit satisfaction rating. Of the 2,986 total telecoaching visits completed, 54% (*n* = 1,621 visits) were rated. Average satisfaction was 4.88 (SD = 0.36, range: 2–5) for the full sample and similar in those with elevated baseline depressive-related (mean [M] = 4.88, SD = 0.35) and burnout symptoms (M = 4.87, SD = 0.38).

## Discussion

We evaluated the real-world effectiveness of evidence-based telecoaching in people seeking mental health services through an employer-sponsored benefit during the COVID-19 pandemic. We examined changes in mental health and workplace outcomes before and after telecoaching in employees. We found that well-being significantly improved, while presenteeism and absenteeism significantly decreased in the full sample, but all changes were of negligible effect sizes. This indicates that telecoaching may improve mental health concerns that underlie patterns of workplace behavior and productivity. Well-being and burnout significantly improved among those with elevated symptoms, which were of a large and small effect size, respectively. Visit utilization was found to be a significant moderator of changes in well-being, absenteeism, and presenteeism; employees who had utilized 2 or more telecoaching visits reported significant improvements in outcomes, while those who had utilized less remained unchanged. Satisfaction with telecoaching was high (average of 4.88/5), indicating favorable treatment acceptability and therapeutic alliance. Thus, telecoaching may be an effective way to enhance mental health care delivery by facilitating positive outcomes and workplace productivity during times of crisis.

We observed statistically significant improvements in well-being and burnout in participants beginning care with elevated baseline symptoms, as well as higher rates of clinically relevant change and reduction from entry. These findings align with previous coaching research in the general workforce^23,40^ and in employees with existing health conditions.^41^ The rate of clinically relevant change in well-being we observed in our full sample (25.6%) was higher than those previously in the other telemental health interventions (13%).^42^ Our results did follow a similar pattern of previous work showing greater effectiveness in those with higher baseline symptoms. This may indicate that evidence-based telecoaching is more beneficial for people with higher mental health needs than people with higher well-being or lower burnout at entry. It is possible that people who start higher in well-being may experience a ceiling effect, and may benefit more from lighter touch interventions such as digital CBT, or that other outcomes are more relevant for them.

We also found that telecoaching visit utilization moderated changes in well-being, absenteeism, and burnout such that participants generally reported greater improvements with more sessions. This supports our hypothesis and confirms prior work showing that more coaching sessions are beneficial for coping and self-regulation.^20^ Future research should explore factors associated with higher visit utilization in real-world settings, such as care preferences and the coaching relationship. For example, some participants may have preferred to receive care through group sessions or self-guided resources. Supporting individuals' decision-making and care preferences may increase utilization and improve outcomes.^43^

People may have utilized more visits when they felt more trust with their coach, driving greater improvements in well-being. A recent meta-analysis found that working alliance (quality of the coaching relationship) was associated with positive outcomes.^44^ However, other research suggests that the strength of the working alliance only correlates with higher coaching effectiveness at the beginning of care and not as care progresses.^45^ Measuring working alliance as a predictor of utilization and a moderator of outcomes is important for future research.

We did not find that burnout statistically improved in the full sample, which differs from previous research.^20^ Our findings may be unique because we exclusively analyzed records from participants who initiated telecoaching during the COVID-19 pandemic. Thus, variables not captured here, such as national trends in elevated distress during the pandemic,^4–6^ may have contributed to this lack of observed improvement, as well as the significant but negligible observed effect sizes for well-being, presenteeism, and absenteeism. Yet, our results also revealed that these outcomes did not worsen, indicating that evidence-based telecoaching may be beneficial in helping individuals maintain their mental health and productivity during distressing times. Thus, telecoaching for mental health is an important investment to support employee well-being with potential for cascading positive effects on employee productivity.

Regarding limitations, our cohort design lacked a comparison group or random assignment and our inclusion/exclusion criteria were constrained by available data reducing the ability to draw causal inferences. Because we did not utilize a randomized controlled trial design, we cannot be sure that the results obtained were due to the intervention, as opposed to the passage of time or regression to the mean. Additional research with an experimental design is needed to confirm our results. We were also limited by the outcome data available: of the 15,119 participants who initiated telecoaching during the study period, only 1,228 provided valid assessments. The participants who did not provide assessments had similar visit utilization rates to those who did (2.7 vs. 2.4), which should encourage employers that telecoaching services provided through a mental health benefit are utilized. However, a significant limitation of our complete-case analysis is the potential for bias in our estimates and the increased likelihood that our results may not generalize to all individuals who engage with telecoaching. Within our analysis sample, there was also some degree of missing follow-up data for three of our outcomes. Missing data are more common in real-world settings (e.g., chart review of routine care) compared to structural clinical trials. Even though data were missing completely at random and may not bias results, future studies should implement procedures (such as prompts or incentives) to capture more complete follow-up data. In addition, the use of employees' self-report of absenteeism and presenteeism in our analysis may be subject to information bias, which future studies may be able to address by seeking to incorporate objective data provided by employers to mitigate. Future research could also consider an equivalence trial to determine the comparative effectiveness of telecoaching and teletherapy for individuals with subclinical symptoms.

## Conclusions

This study demonstrated the real-world effectiveness of evidence-based telecoaching delivered as part of an employer-sponsored mental health benefits platform. Leveraging videoconferencing, telecoaching had positive effects on mental health and workplace outcomes during the COVID-19 pandemic. Telecoaching may have similar effectiveness as face-to-face coaching^46^ and is well-suited to provide employees access to care remotely.^47^ Thus, telecoaching represents a vital option for achieving optimal outcomes in people who need mental health services.

## Data Sharing

The data used in this study are not publicly available or accessible.
